# A Network-Based Approach to Investigate the Neuroprotective Effects and Mechanisms of Action of Huangqi-Chuanxiong and Sanleng-Ezhu Herb Pairs in the Treatment of Cerebral Ischemic Stroke

**DOI:** 10.3389/fphar.2022.844186

**Published:** 2022-03-23

**Authors:** Lin Zhao, Li Dong Ding, Zi Hao Xia, Peng Sheng, Meng Meng Shen, Zhong Ming Cai, Bing Chun Yan

**Affiliations:** ^1^ Medical College, Institute of Translational Medicine, Yangzhou University, Yangzhou, China; ^2^ Jiangsu Key Laboratory of Integrated Traditional Chinese and Western Medicine for Prevention and Treatment of Senile Diseases, Yangzhou University, Yangzhou, China; ^3^ Department of Neurology, Taizhou Second People's Hospital, Taizhou, China; ^4^ Department of Neurology, Yangzhou Hospital of Chinese Medicine, Yangzhou, China

**Keywords:** huangqi-chuanxiong, sanleng-ezhu herb, ischemic stroke, clinical data mining, network pharmacology

## Abstract

**Objective:** We aimed to investigate the effect and mechanisms of action of two drug pairs [Huangqi-Chuanxiong and Sanleng-Ezhu Herb (HCSE)] on the treatment of ischemic stroke.

**Materials and methods:** We mined the current literature related to ischemic stroke and formulated a new formulation of Chinese herbs. Then, we identified the main candidate target genes of the new formulation by network pharmacology. Next, we performed enrichment analysis of the target genes to identify the potential mechanism of action of the new formulation in the treatment of ischemic stroke. Next, we experimentally validated the mechanism of action of the new formulation against ischemic stroke. Infarct volume and neurological deficits were evaluated by 2,3,5-triphenyltetrazolium (TTC) staining and Longa’s score, respectively. The predicted pathways of signal-related proteins were detected by western blotting.

**Results:** We mined the current literature and identified a new formulation of Chinese herbs for the treatment of ischemic stroke. The formulation included Huangqi, Chuanxiong, Sanleng and Ezhu. Next, we used network pharmacological analysis to identify 23 active compounds and 327 target genes for the new formulation. The key target genes were *MAPK3*, *MAPK1*, *HSP90AA1*, *STAT3*, *PIK3R1*, *PIK3CA* and *AKT1*. Kyoto Encyclopedia of Genes and Genomes (KEGG) enrichment analysis revealed significant enrichment of the PI3K/AKT and MAPK/ERK signaling pathways. By performing experiments, we found that the new formulation reduced the infarct volume of middle cerebral artery occlusion (MCAO) induced mice and activated the PI3K/AKT and MAPK/ERK signaling pathways. These findings confirmed that the new formulation has a significant protective effect against ischemic stroke injury by activating the PI3K/AKT and MAPK/ERK signaling pathways.

**Conclusion:** We identified a new treatment formulation for ischemic stroke by data mining and network pharmacological target prediction. The beneficial effects of the new formulation act by regulating multiple target genes and pathways. The mechanism of action of the new formulation may be related to the AKT and ERK signaling pathways. Our findings provide a theoretical basis for the effects of the new formulation on ischemic stroke injury.

## Introduction

Stroke has become the second leading cause of global death and is the leading cause of death and disability among adults in China. Ischemic stroke accounts for 69.6–77.8% of stroke cases and is associated with mortality and recurrence rates of 7 and 16%, respectively (Wang et al., 2017; Yiping Chen et al., 2020). Ischemic stroke causes irreversible damage to the brain tissue by influencing the distribution of affected blood vessels, thus leading to a variety of neurological symptoms and signs. Ischemic stroke adversely affects the health of patients and places significant socioeconomic burden on their families (Mukundan and Seidenwurm, 2018).

According to the theory of traditional Chinese medicine (TCM) (Liu et al., 2007), the main syndromes and causes of ischemic stroke are Qi deficiency and blood stasis syndrome (Li et al., 2014; Zhai et al., 2020). Therefore, the main principle of treatment for ischemic stroke is the invigoration of Qi and the promotion of blood circulation (Yu Wang et al., 2020). Based on this principle, practitioners of ancient Chinese medicine have suggested many treatments for stroke (Xu et al., 2019). The most commonly used and most effective treatment is Buyang Huanwu Decoction, invented by Wang Qingren, a famous doctor in the Qing Dynasty. Several studies have shown that the modified Buyang Huanwu Decoction has significant positive effects on the neurological deficits of patients with ischemic stroke (Xi Chen et al., 2020). When using Buyang Huanwu Decoction, the effect of invigoration of Qi and the promotion of blood circulation is mainly induced by two specific drugs: Huangqi and Chuanxiong. Some drugs mainly treated blood stasis, such as Taoren (Xi et al., 2013). However, research on the molecular effects associated with the invigoration of Qi and the promotion of blood circulation is scarce. Therefore, in the present study, we investigated the molecular mechanisms underlying the traditional methods used to prevent and treat ischemic stroke.

Data mining and network pharmacology can systematically evaluate the interactions between diseases and drugs and identify the specific mechanisms of action of drugs on their gene targets. Many previous studies have used data mining and network pharmacology to investigate the treatment of diseases by TCM (Li and Zhang, 2013; Sun et al., 2020). These strategies can provide new ideas for research and allow for the more accurate application of TCM.

In the present study, data mining and network pharmacology were used to evaluate the related mechanisms and effects of Huangqi-Chuanxiong and Sanleng-Ezhu Herb pairs (HCSE) on the treatment of patients suffering from ischemic stroke with Qi deficiency and blood stasis syndrome. Our findings provide scientific evidence to support the application of the new formulation for the treatment of ischemic stroke.

## Materials and Methods

### Data Mining

#### Literature Review

First, we searched the China National Knowledge Infrastructure, Wanfang Data, and PubMed using the keywords “stroke” and “ischemic stroke with Qi deficiency and blood stasis syndrome” to identify articles published the 1st September 2011 and the 1st September 2021. Then, we used the keywords “circulating blood and removing stasis” and “broking blood stasis” to search the clinical literature on the use of TCM to treat ischemic stroke with Qi deficit and blood stasis syndrome (Liu et al., 2012).

### Data Screening

The prescriptions included in the retrieved literature were analyzed, and matrix distribution was performed.

### Association Analysis

The drugs were analyzed using the *a priori* module included in SPSS Modeler software (version 18.0; IBM Corp., Armonk, NY, USA). The minimum number of conditional supports, indicating the number of drug combinations in the prescriptions, was set to 10. The minimum rule confidence, indicating the probabilities of occurrence for the first and second terms of the rule, was set to 80%. Drug pairs were then identified based on the association rules, and the new formulations were combined using TCM theory analysis.

### Network Pharmacology

#### Prediction of Target Genes for the Drug Ingredients

The active compounds of the drugs were screened based on an oral bioavailability (OB) ≥ 30%, a drug-likeness (DL) ≥ 0.18, and a blood-brain barrier (BBB) ≥ −0.3 using the TCMSP database (https://tcmsp-e.com/) (Ru et al., 2014). Target proteins of the active ingredients were identified using SwissTargetPrediction (Swiss Institute of Bioinformatics, Basel, Switzerland; http://www.swisstargetprediction.ch/) (Daina et al., 2019).

#### Disease Target Identification

The keyword “ischemic stroke” was used to search disease targets in the GeneCards database (https://www.genecards.org/) (Safran et al., 2021). According to the principles of TCM, the main symptoms of ischemic stroke with Qi deficiency and blood stasis syndrome are a pale white complexion, a shortness of breath, palpitations, spontaneous sweating, loose stools, limb swelling, angular salivation, a purple tongue, a thin and whitish tongue coating, and a deep and faint pulse. We used these keywords as search terms in the GeneCards database. These symptoms were then selected as the targets for Qi deficiency and blood stasis syndrome. Overlapping targets were considered as potential targets of ischemic stroke with Qi deficiency and blood stasis syndrome.

#### Predicting Overlapping Drug and Disease Targets

Drug and disease targets were imported into Jvenn software (http://jvenn.toulouse.inra.fr/app/example.html) (Bardou et al., 2014) to construct a Venn diagram; overlapping targets were then considered to be potential therapeutic targets for the new treatment for ischemic stroke.

#### Construction of a Protein-Protein Interaction Network

A PPI network was constructed for the potential therapeutic target proteins of the new treatment formulation using the STRING database (https://string-db.org). The results were then visualized using Cytoscape software (version 3.8.2; https://cytoscape.org/). The degree of freedom was indicated by node size and color. The topological properties of the target genes were analyzed using the CytoNCA tool (Cytoscape software plugin) (Tang et al., 2015; Bei Yin et al., 2020). The PPI network was used to screen key targets of the new treatment based on a degree centrality (DC) > two-fold of the median and betweenness centrality (BC), closeness centrality (CC), and an eigenvector centrality (EC) > one-fold of the median. Higher quantitative values were correlated with a greater importance of the node.

#### Gene Ontology Enrichment and Kyoto Encyclopedia of Genes and Genomes Pathway Analyses

Metascape (https://metascape.org/) was used to perform GO and KEGG pathway enrichment analyses of 327 candidate target genes, according to the molecular function (MF), biological process (BP), and cellular component (CCT) categories (Zhou et al., 2019). *p* values were used to evaluate the proteins each GO annotation, thus reflecting the significance of the biological function. In addition, the FDR error control method (FDR <0.05) was used to test and correct the *p* value. The threshold value of *p* < 0.05 was finally used to screen the biological processes with significant differences. GO and KEGG enrichment analyses were visualized by the Bioinformatics platform (http://www.bioinformatics.com.cn/).

### Experimental Verification

#### Experimental Animals

Healthy male Institute of Cancer Research mice (ICR; body weight: 32–35 g) were provided by the Comparative Medicine Center of Yangzhou University (Yangzhou, China) and used after 1 week of acclimation. The mice were housed in a controlled condition with a 12-h light/dark cycle at 23°C and 60% humidity with free access to food and water. All experimental investigation procedures for animals were permitted by the Yangzhou University Institutional Animal Care and Use Committee (Grant No. YIACUC-14-0015).

#### Middle Cerebral Artery Occlusion

The middle cerebral artery occlusion (MCAO) model was created using the following procedure. The mice were continuously anesthetized by the inhalation of a mixture of 30% oxygen, 70% nitrogen and 3–4% isoflurane (RWD Life Science, Guangdong Province, China). The mice were then fixed in the supine position. Next, we sterilized and dissected the skin of the middle of the neck. The left common carotid artery (CCA), the internal carotid artery (ICA), and the external carotid artery (ECA), were then separated with a blunt instrument. The ECA was ligated, the CCA and ICA were clamped with a bulldog clip, and a “V” incision was made in the ECA with ophthalmic scissors. A 0.23 mm monofilament nylon suture (Beijing Biotechnology Co., Ltd.) was then inserted through the “V” incision into the ICA until slight resistance was achieved. After 45 min of arterial occlusion, the monofilament nylon suture was removed, and the blood was reperfused for 24 h. In the Sham group, only the CCA, ICA and ECA were separated; no monofilament nylon suture was inserted. The body temperature of the mice was maintained at 37.0–37.5°C during surgery.

#### Experimental Groups and Drug Treatments

The mice were randomly divided into four groups as follows (*n* = 21 in each group): 1) a control group and 2) a pre-HCSE (13.65 g/kg) group. Each of these two groups was then further divided into a Sham group and a MCAO group. All animals were killed at 24 h after cerebral ischemia reperfusion. The method used to prepare HCSE involved mixing 225 g of Huangqi (100 g), Chuanxiong (50 g), Sanleng (45 g), and Ezhu (30 g) with eight times the volume of distilled water, followed by boiling for 2 h. Then, the filtrate was collected, and the filtrate was extracted again with three times the volume of distilled water. The filtrate was mixed twice and concentrated to 225 ml by a rotary evaporator. Therefore, the final crude drug concentration was 1 g/ml. All herbs were purchased from the Yangzhou Hospital of Chinese Medicine, Jiangsu Province, China. According to the dose used for ischemic stroke patients in the clinic (90 ml per day), we determined the dose for our experimental animals by extrapolation from the human dose in accordance with a previous study (Reagan-Shaw et al., 2008). Finally, a dose of 13.65 g/kg was obtained for administration. We chose 6.83 g/kg, 13.65 g/kg, and 27.3 g/kg as the HCSE doses in our preliminary study. We found that the neuroprotective effects (as determined by TTC staining) of the 13.65 g/kg and 27.3 g/kg doses were better than those with the 6.83 g/kg dose. Therefore, we selected a dose of 13.65 g/kg for use in the present study.

HCSE was administered intra-gastrically the same day, 24 h, and 48 h before ischemic surgery, and was administered twice a day. The Sham group and the ischemia group received the same amount of 0.9% saline intra-gastrically for the same durations. Following the last administration, the mice underwent ischemia/reperfusion or Sham operation.

#### Neurological Deficit Assessment

The degree of neurological deficit was assessed 24 h after reperfusion using the Longa score, as follows: 1) no neurological deficit: 0 points; 2) inability to fully extend the front paw on the paralyzed side: 1 point; 3) circling to the paralyzed side during walking: 2 points; 4) leaning towards the paralyzed side when walking: 3 points; and 5) inability to walk spontaneously, with loss of consciousness: 4 points. A score of more than 1 indicates that the MCAO model had been successfully established.

#### TTC Staining and the Quantification of Infarct Volume

Next, we performed TTC staining experiments with reference to relevant published literature (Liu et al., 2020; Zhang et al., 2021). After 24 h of ischemia/reperfusion, mice were anesthetized by isoflurane inhalation and killed by cervical dislocation. Brain tissue was then removed and cut into 2 mm thick coronal sections. The sections were then stained using 2% 2,3,5-triphenyltetrazolium chloride (Sigma-Aldrich, St. Louis, MO) in the dark at 37.0°C for 30 min. After staining, the brain sections were fixed in 4% paraformaldehyde buffer. The live portion of the brain section was red, and the infarcted portion was pale white. The infarct volume and whole volume of each brain slice were measured using Image Pro Plus 6.0 software (Media Cybernetics, Bethesda, MD, USA). The infarct volume of each section was multiplied by the layer thickness (2 mm) to calculate the total infarct volume. The ratio of total infarct volume/whole brain volume × 100% was used as the total infarct ratio.

### Western Blot Analysis

Western blotting was carried out as described in our previous article (Liu et al., 2021). First, hippocampal and cortical tissues were lysed with a Whole Protein Extraction Kit (Solarbio, Beijing, China). The protein concentration of each sample was then measured using a BCA protein assay kit (Vazyme, Nanjing, China). Equal amounts of protein (30 μg) were then separated by 10% sodium dodecyl sulfate polyacrylamide gel electrophoresis (SDS-PAGE) and transferred to nitrocellulose membranes (Millipore, Bedford, USA). The membranes were cultured in 5% BSA in TBS containing 0.1% Tween 20 for 90 min to prevent non-specific protein binding sites from binding to antibodies. The membranes were cut according to molecular weight and then incubated overnight at 4°C with rabbit anti-AKT (1:1,000, Cell Signaling Technology), rabbit anti-p-AKT (1:2000, Cell Signaling Technology), rabbit anti-ERK 1/2 (1:1,000, Cell Signaling Technology), rabbit anti-p-ERK 1/2 (1:1,000, Cell Signaling Technology). Subsequently, the membrane was incubated with a corresponding secondary antibody at room temperature for 2 h. The protein membranes were then to Super Signal West Pico chemiluminescence substrates (CWBIO, Beijing, China) and observed by Fluor Chem M (Protein Simple, USA). Western blot results were analyzed by Image Pro Plus 6.0.

### Data Analysis

Data are expressed as a mean ± standard deviation (‾*X* ± SD) and analyzed by GraphPad Prism 8.0. SPSS software (version 21.0; IBM, Armonk, NY, USA). The student’s two-tailed *t*-test was used for comparisons between two groups and one-way analysis of variance followed by Dunnett’s test was used for the comparison of three or more groups. *p* < 0.05 was considered statistically significant.

## Results

### Data Mining

#### Literature Analysis

We identified 177 articles related to TCM treatment of ischemic stroke with Qi deficiency and blood stasis syndrome, including 76 prescriptions based on 119 TCM drugs. In total, 77 articles were related to breaking blood and removing blood stasis for the treatment of ischemic stroke with Qi deficiency and blood stasis syndrome; these articles included 52 prescriptions based on 94 TCM drugs.

#### Types of TCM Drugs

The 10 most common TCM drugs used for the treatment of ischemic stroke with Qi deficiency and blood stasis syndrome were Huangqi (*n* = 69), Chuanxiong (*n* = 64), Honghua (*n* = 51), Danggui (*n* = 50), Dilong (*n* = 50), Chishao (*n* = 46), Taoren (*n* = 40), Danshen (*n* = 27), Shuizhi (*n* = 20), Jixueteng (*n* = 19), and Sanqi (*n* = 14). The 10 most common TCM drugs used for breaking blood and removing stasis were Chuanxiong (*n* = 46), Honghua (*n* = 39), Sanleng (*n* = 37), Ezhu (*n* = 37), Danshen (*n* = 35), Danggui (*n* = 35), Chishao (*n* = 29), Taoren (*n* = 29), Huangqi (*n* = 26), and Dilong (*n* = 19).

#### Association Analyses

The five most commonly used drug combinations for TCM treatment of ischemic stroke with Qi deficiency and blood stasis syndrome were Chuanxiong/Huangqi, Huangqi/Chuanxiong, Chishao/Honghua, Dilong/Honghua, and Danggui/Honghua (Table 1). The five most commonly used drug combinations for breaking blood and removing blood stasis for the treatment of ischemic stroke with Qi deficiency and blood stasis syndrome were Ezhu/Sanleng, Sanleng/Ezhu, Honghua/Danggui, Chuanxiong/Danggui, and Chuanxiong/Taoren (Table 2). Based on these results, we decided to combine Huangqi, Chuanxiong, Sanleng, and Ezhu to form a new formulation (HCSE).

**TABLE 1:** Drug associations.

Serial number	Drug pair	Support (%)	Confidence (%)
1	Chuanxiong/Huangqi	90.79	86.96
2	Huangqi/Chuanxiong	84.21	93.75
3	Chishao/Honghua	67.11	82.35
4	Dilong/Honghua	67.11	86.27
5	Danggui/Honghua	67.11	88.23

**TABLE 2:** Drug associations.

Serial number	Drug pair	Support (%)	Confidence (%)
1	Ezhu/Sanleng	53.62	86.49
2	Sanleng/Ezhu	52.17	88.89
3	Honghua/Danggui	50.72	85.71
4	Chuanxiong/Danggui	50.72	80.00
5	Chuanxiong/Taoren	42.01	86.21

### Network Pharmacology

#### Chemical Component Analysis

After excluding components with undiscovered targets, 26 candidate components of Huangqi, Chuanxiong, Sanleng, and Ezhu were identified in the TCMSP database based on specific thresholds (OB ≥ 30%, DL ≥ 0.18, and BBB ≥ −0.3). In total, 12, 5, 5, and 3 compounds were identified from Huangqi, Chuanxiong, Sanleng and Ezhu, respectively (Table 3). Of these, Huangqi, Sanleng and Ezhu all contain Hederagenin as a key ingredient; Huangqi and Sanleng both contain Formononetin as a key ingredient.

**TABLE 3:** Active compounds contained in the new formulation.

Serial number	Herb	Active component	Mol ID	OB	DL	BBB
1	Huangqi	Betulinic acid	MOL000211	55.38	0.78	0.22
2	Huangqi	Kumatakenin	MOL000239	50.83	0.29	−0.22
3	Huangqi	Hederagenin	MOL000296	36.91	0.75	0.96
4	Huangqi	Beta-sitosterol	MOL000033	36.23	0.78	1.09
5	Huangqi	3,9,10-Trimethoxypterocarpan	MOL000371	53.74	0.48	0.63
6	Huangqi	7-O-Methylisomucronulatol	MOL000378	74.69	0.3	0.84
7	Huangqi	Astrapterocarpan	MOL000380	64.26	0.42	0.55
8	Huangqi	Bifendate	MOL000387	31.1	0.67	−0.06
9	Huangqi	Formononetin	MOL000392	69.67	0.21	0.02
10	Huangqi	Isoflavanone	MOL000398	109.99	0.3	0.17
11	Huangqi	Isomucronulatol 7-O-glucoside	MOL000438	67.67	0.26	0.34
12	Huangqi	3,4-(4-methoxy-6-hydroxy-1,2-phenyleneoxy)-5-hydroxy-7-methoxy-2H-1-benzopyran	MOL000442	39.05	0.48	−0.04
13	Chuanxiong	Ethyl linoleate	MOL001494	42	0.19	1.14
14	Chuanxiong	Myricanone	MOL002135	40.6	0.51	−0.08
15	Chuanxiong	Perlolyrine	MOL002140	65.95	0.27	0.15
16	Chuanxiong	Senkyunone	MOL002151	47.66	0.24	0.5
17	Chuanxiong	Wallichilide	MOL002157	42.31	0.71	0.73
18	Chuanxiong	3-Epi-beta-sitosterol	MOL000359	36.91	0.75	0.87
19	Sanleng	trans-11-eicosenoic acid	MOL001297	30.7	0.2	0.89
20	Sanleng	Hederagenin	MOL000296	36.91	0.75	0.96
21	Sanleng	beta-Sitosterol	MOL000358	36.91	0.75	0.99
22	Sanleng	Formononetin	MOL000392	69.67	0.21	0.02
23	Sanleng	Stigmasterol	MOL000449	43.83	0.76	1
24	Ezhu	Hederagenin	MOL000296	36.91	0.75	0.96
25	Ezhu	Wenjine	MOL000906	47.93	0.27	0.3
26	Ezhu	Bisdemethoxycurcumin	MOL000940	77.38	0.26	−0.08

#### Integration of Disease and Drug Target Genes

**FIGURE 1:**
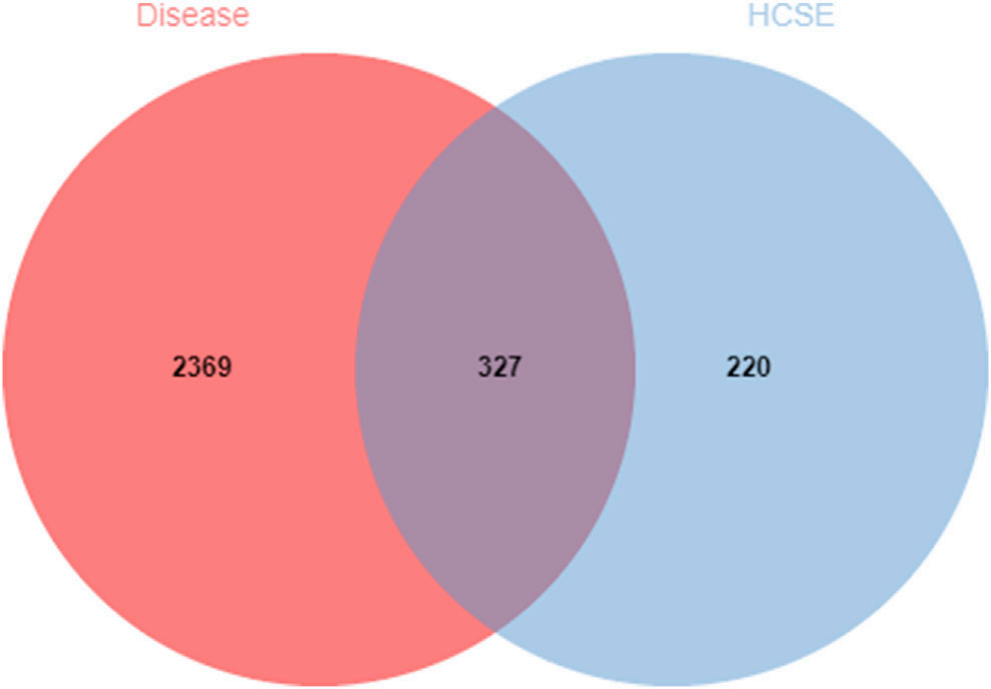
Venn diagram of the overlapping drug and disease target genes. The red circle represents the targets for ischemic stroke with Qi deficiency and blood stasis syndrome. The blue circle represents the targets for the new formulation. The 327 overlapping genes are potential therapeutic targets for the new formulation against ischemic stroke with Qi deficiency and blood stasis syndrome.

The GeneCards database was used to integrate 3,854 genes related to ischemic stroke and 5,103 genes related to the syndrome of Qi deficiency and blood stasis syndrome; these analyses identified a total of 2,696 genes. Then, the target genes of the drug components and disease were compared in a Venn diagram using Jvenn software. The Venn diagram showed that 327 genes were potential targets of the new formulation for the treatment of ischemic stroke with Qi deficiency and blood stasis syndrome (Figure 1).

#### PPI Network

**FIGURE 2:**
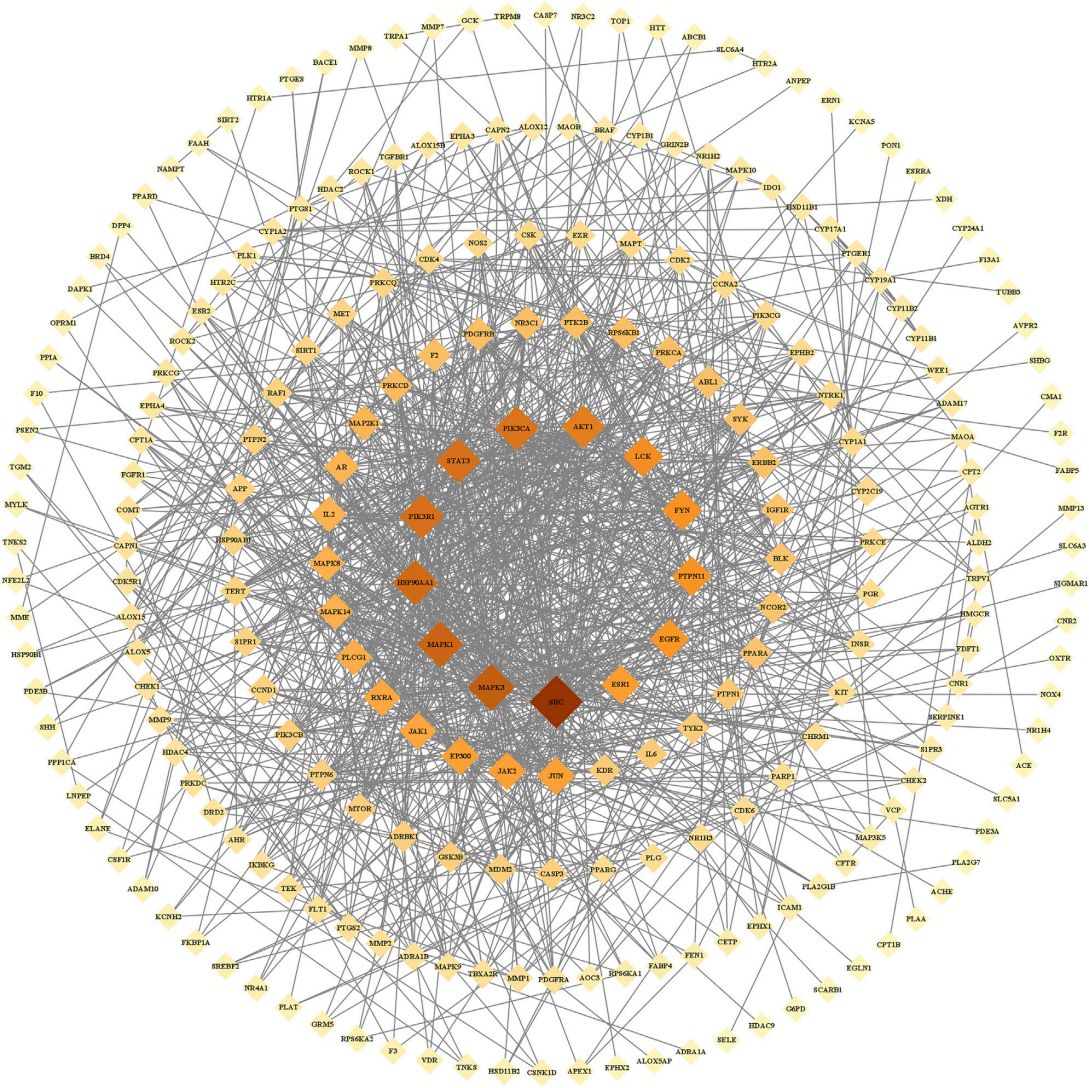
Protein-protein interaction network of the core proteins targeted by the new formulation for the treatment of ischemic stroke with Qi deficiency and blood stasis syndrome. The network included 244 nodes and 1,081 edges. Diamonds repr6+ the target proteins, with darker colors indicating increasing importance.

**FIGURE 3:**
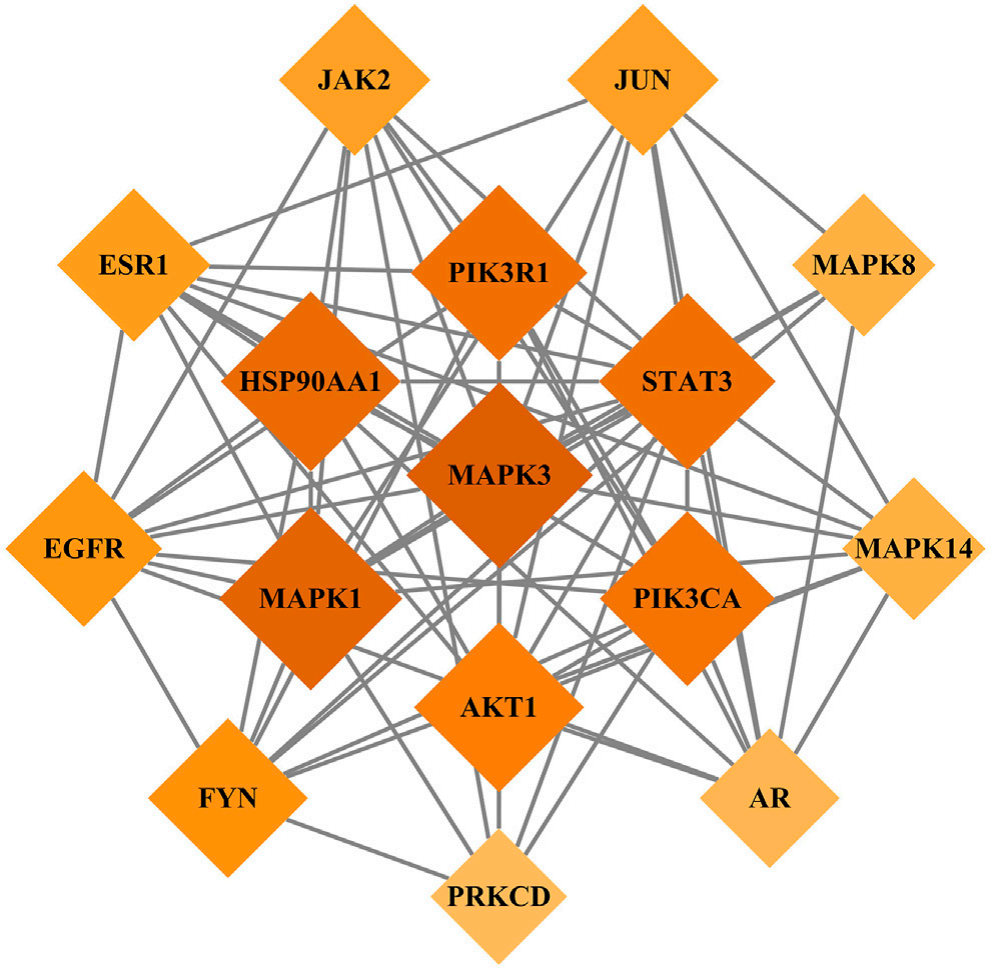
Key target genes of the new treatment formulation for ischemic stroke with Qi deficiency and blood stasis syndrome. Diamonds represent target genes, with darker colors indicating increasing importance.

We imported the 327 potential therapeutic target genes into the STRING database. Then, we imported target genes with a confidence >0.9 into Cytoscape 3.8.2 software to construct a PPI network. In the PPI network, the nodes represent the target genes, and the node size and color represent the degrees of freedom. As shown in Figure 2, the network consisted of 244 nodes and 1,081 edges. The central properties of the nodes were estimated by topological analysis. Sixteen key gene targets of the new formulation for the treatment of ischemic stroke with Qi deficiency and blood stasis syndrome were screened using the following criteria: DC > 11, BC > 728.62274, CC > 0.37587029, and EC > 0.09713785. In Figure 3, the size of the nodes is proportional to the DC. Notably, MAPK3, MAPK1, HSP90AA1, STAT3, PIK3R1, PIK3CA, and AKT1 were the main target proteins involved in the pathogenesis of ischemic stroke with Qi deficiency and blood stasis syndrome. Among these targets, MAPK3 (degree = 50) was shown to be the most important protein in the PPI network.

#### GO Enrichment Analysis

**FIGURE 4:**
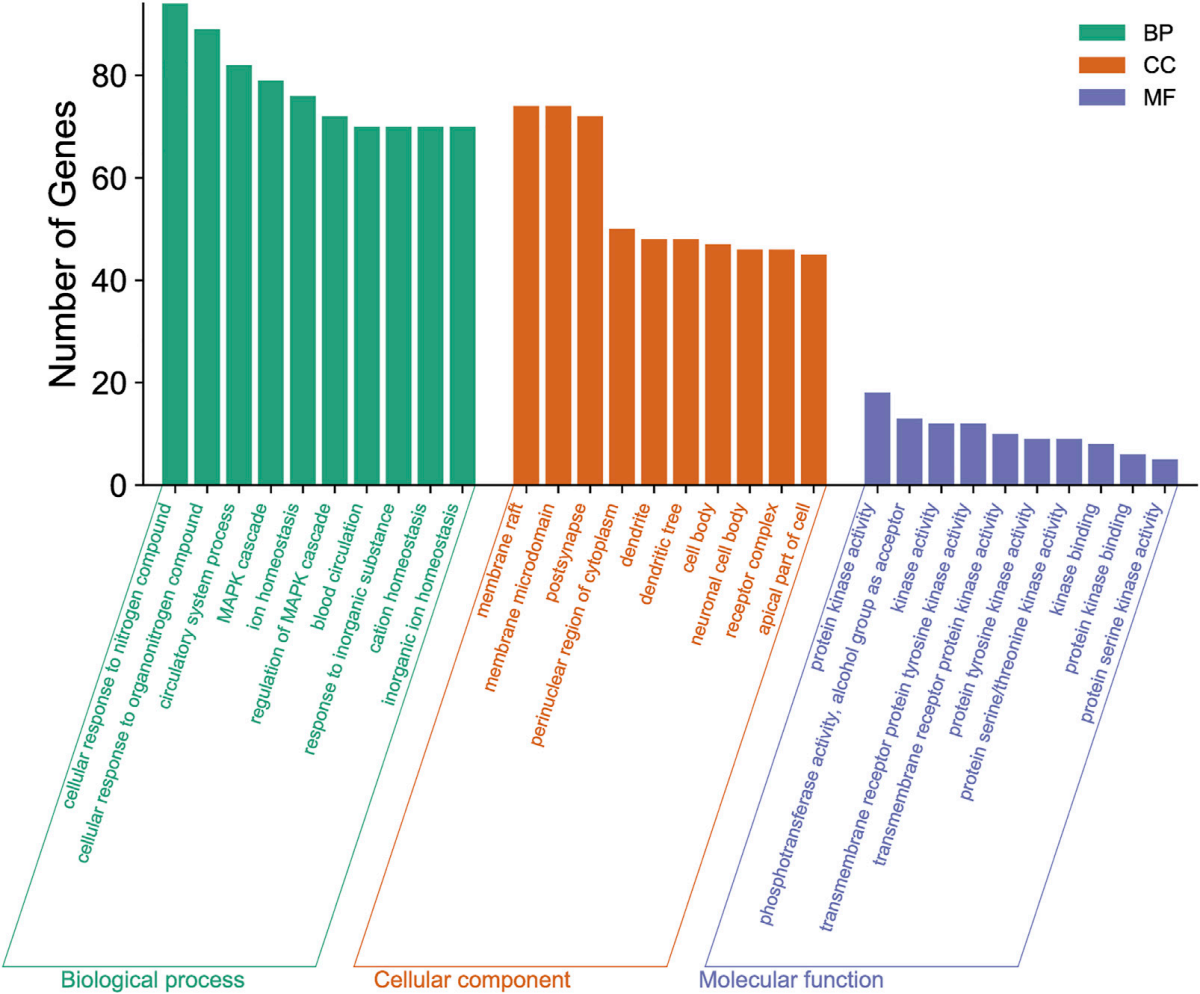
Gene Ontology (GO) enrichment analysis of the new treatment formulation for ischemic stroke with Qi deficiency and blood stasis syndrome. The top 10 biological processes, cellular components, and molecular functions are shown as a bar chart. The *x*-axis represents the GO terms and the *y*-axis represents the number of annotated genes.

To explore the functional distribution of the gene targets for the formulation, we imported the 327 predicted target genes into the Metascape database for GO enrichment analysis. These genes were found to be associated with multiple BP, CCT, and MF categories (*p* < 0.01; Figure 4). The main BP terms were involved in cellular response to nitrogen compound, cellular response to organonitrogen compound, circulatory system processes, the MAPK cascade, and ion homeostasis. The CCT terms included membrane rafts, membrane microdomains, post-synapse, the perinuclear region of the cytoplasm, and dendrites. The MF terms included protein kinase activity, phosphotransferase activity, alcohol group as acceptor kinase activity, transmembrane receptor protein tyrosine kinase activity, and transmembrane receptor protein kinase activity.

#### KEGG Pathway Annotation

**FIGURE 5:**
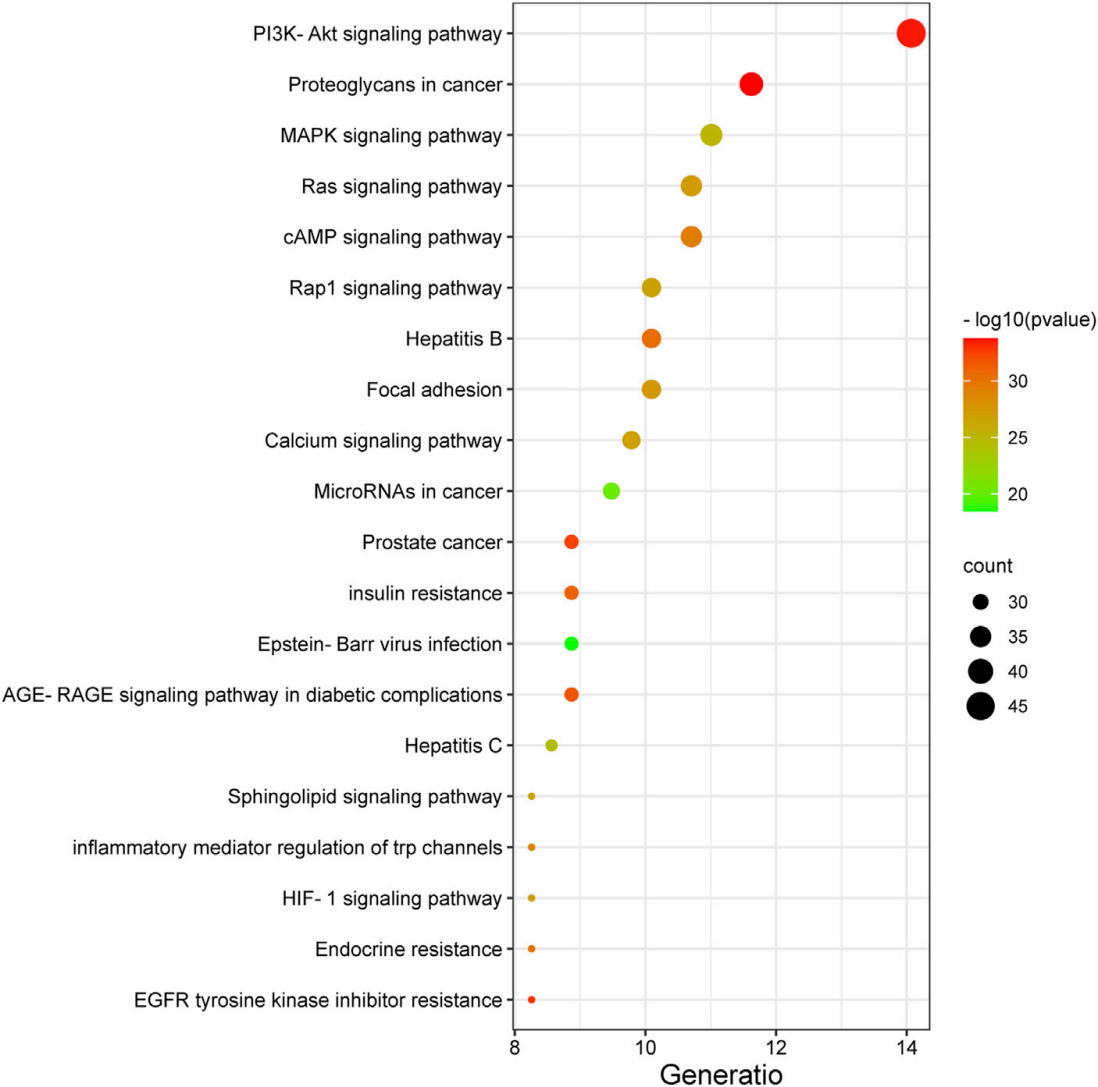
The top 20 pathways identified by KEGG analysis of the new treatment formulation for ischemic stroke with Qi deficiency and blood stasis syndrome. The *y*-axis represents the name of each pathway, and the size of the dots corresponds to the number of genes annotated in the pathway. The *x*-axis represents the ratio corresponding to the significance of the differential protein enrichment, with higher ratios indicating higher reliability.

The 327 potential target genes were imported into the Metascape database for KEGG pathway enrichment analysis. Analysis showed that these targets were enriched in 191 pathways (*p* < 0.01). The top 20 pathways were analyzed using a bioinformatics database (Figure 5). Among these, the PI3K/AKT and MAPK signaling pathways were highly associated with the target genes, thus suggesting that the active ingredients of the new formulation exert beneficial effects on Qi and blood stasis by regulating the PI3K/AKT and MAPK signaling pathways.

### Experimental Verifications

#### The Neuroprotective Effects of HCSE on Ischemic Stroke

**FIGURE 6:**
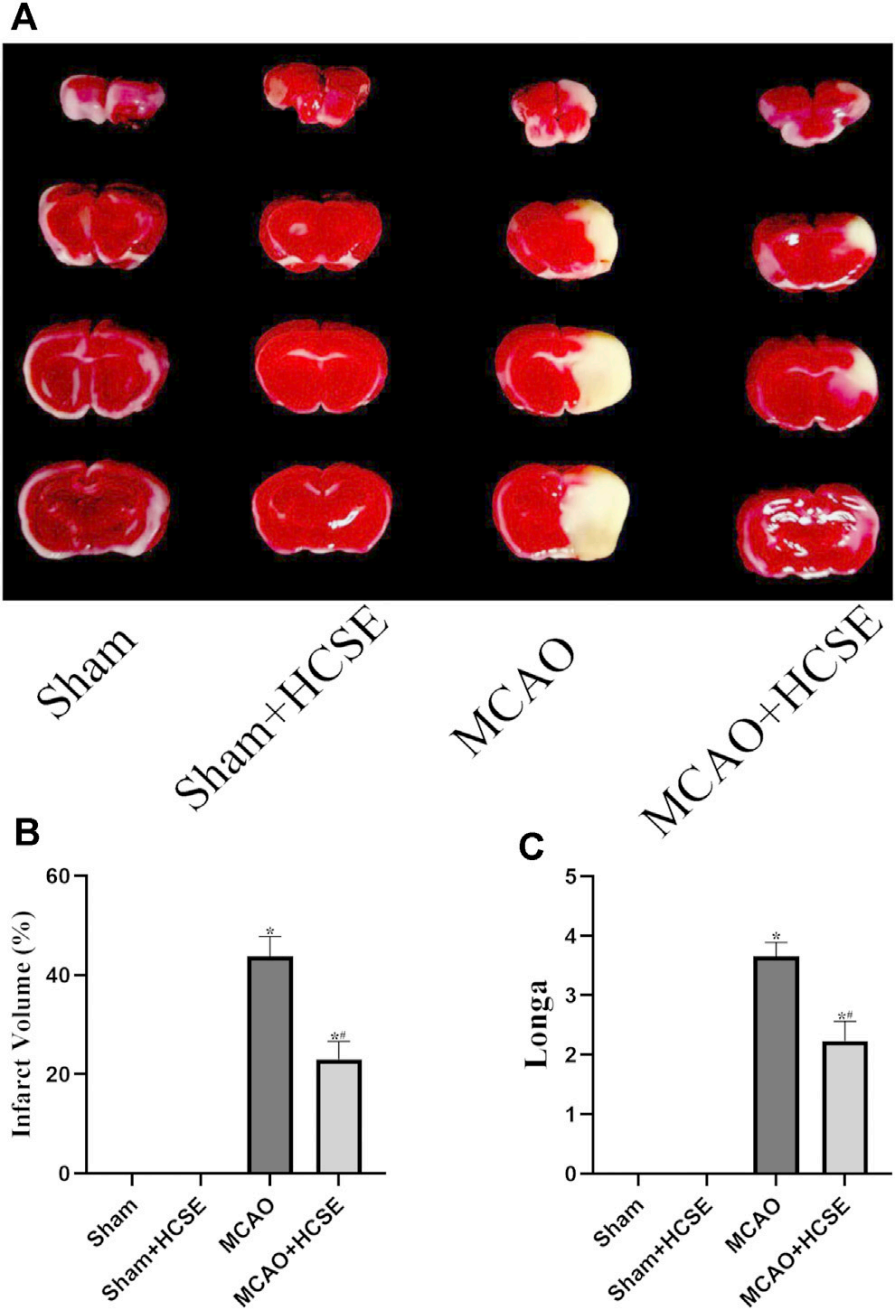
The neuroprotective effects of HCSE. **(A)** TTC staining in the Sham, Sham + HCSE, MCAO and MCAO + HCSE groups. **(B)** The infarct volume was expressed as the ratio of (infarct volume/the whole brain volume) × 100%. **(C)** Longa score. (*n* = 7 per group; *p < 0.05, significantly different from the corresponding Sham group, #*p* < 0.05, significantly different from the MCAO group).

The volume of cerebral infarcts was determined by TTC staining. As shown in Figures 6A,B, no cerebral infarction was seen in the Sham and Sham + HCSE groups. However, the infarct volume in the MCAO group accounted for approximately 44% of the whole brain (*p* < 0.05). Compared with the MCAO group, the infarct volume in the MCAO + HCSE group was significantly smaller (approximately 23% of the brain volume; *p* < 0.05). In addition, the neurological function of the mice was assessed by applying the Longa (0–4) scale. As shown in Figure 6C, no neurological impairment was observed in the Sham and Sham + HCSE groups, although mice in the MCAO group had the highest score (approximately 3.64 points) (*p* < 0.05). Mice in the MCAO + HCSE group had significantly lower neurological function scores (approximately 2.35 points) compared to the MCAO group (*p* < 0.05).

#### The Effect of HCSE on the Levels of AKT/P-AKT/ERK 1/2/p-ERK 1/2 Signaling-Related Proteins in the Hippocampus and Cortex With Ischemic Stroke in Mice

**FIGURE 7:**
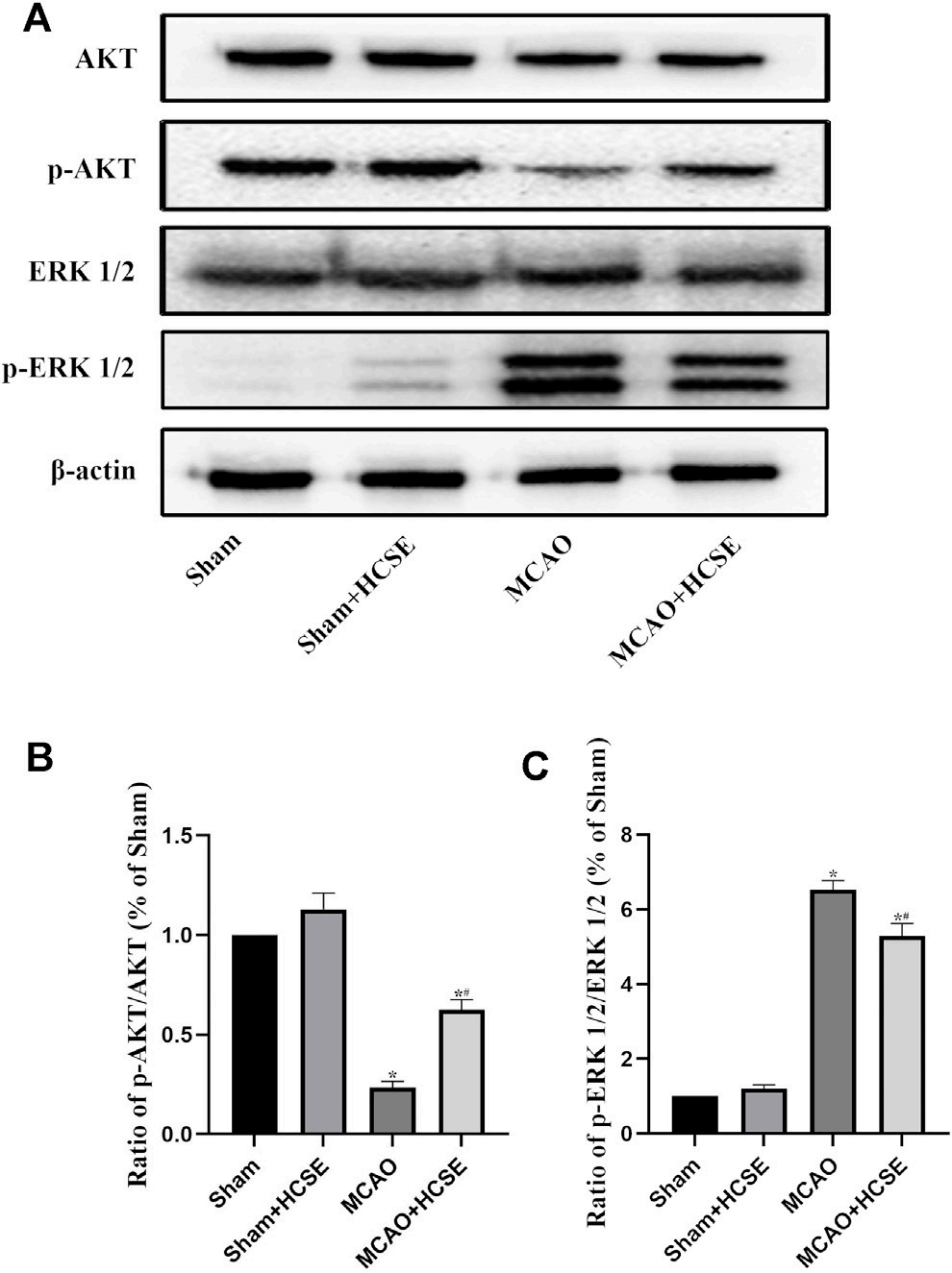
Changes in the levels of proteins related to the AKT/p-AKT/ERK1/2/p-ERK 1/2 signaling pathway in the hippocampus. **(A)** Western blotting of related proteins. **(B)** The ratio of p-AKT/AKT. **(C)** The ratio of p-ERK 1/2/p-ERK 1/2. (*n* = 7 per group; *p < 0.05, significantly different from the corresponding Sham group, #*p* < 0.05, significantly different from the MCAO group).

**FIGURE 8:**
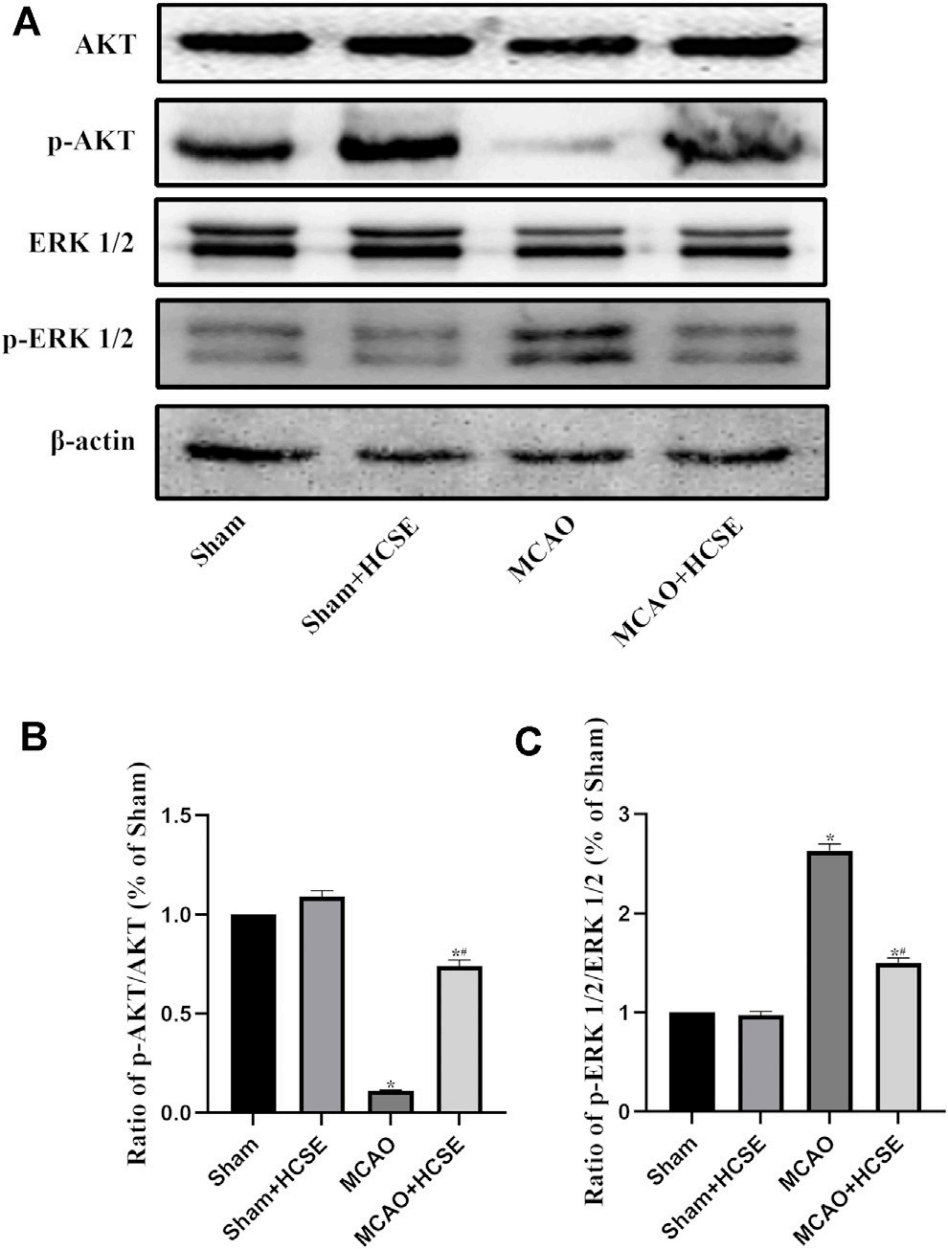
Changes in the levels of proteins related to the AKT/p-AKT/ERK1/2/p-ERK1/2 signaling pathway in the cortex. **(A)** Western blotting of related proteins. **(B)** The ratio of p-AKT/AKT. **(C)** The ratio of p-ERK1/2/p-ERK 1/2. (*n* = 7 per group; *p < 0.05, significantly different from the corresponding Sham group, #*p* < 0.05, significantly different from the MCAO group).

To investigate the possible mechanisms of HCSE against ischemic stroke, we investigated the expression levels of AKT/p-AKT/ERK 1/2/p-ERK 1/2 signaling related proteins by Western blotting. In hippocampal tissue, as shown in Figure 7, there was no significant difference in the p-AKT/AKT and p-ERK 1/2/ERK 1/2 protein ratios in the Sham + HCSE group when compared with the Sham group (*p* < 0.05). The p-AKT/AKT protein ratio in the MCAO group was lower than that in the Sham group (*p* < 0.05), while the p-ERK 1/2/ERK 1/2 protein ratio was higher than that in the Sham group (*p* < 0.05). However, the p-AKT/AKT protein ratio in the MCAO + HCSE group was significantly higher than that in the MCAO group (*p* < 0.05), and the p-ERK 1/2/ERK 1/2 protein ratio was significantly lower than that in the MCAO group (*p* < 0.05). Western blot analyses of cortical tissues revealed the same trend as the hippocampal tissue. As shown in Figure 8, the p-AKT/AKT protein ratio in the MCAO + HCSE group was significantly higher than that in the MCAO group (*p* < 0.05). In addition, the p-ERK 1/2/ERK 1/2 protein ratio was significantly lower than that in the MCAO group (*p* < 0.05).

## Discussion

Although the treatment of stroke is rapidly evolving, research on the use of TCM to treat ischemic stroke is scarce. In the present study, we identified a new treatment formulation for ischemic stroke by the application of data mining and identified the mechanism of action of this treatment formulation using network pharmacology. The new formulation consists of Huangqi, Chuanxiong, SanLeng, and Ezhu. We concluded that the main active ingredients of the new formulation include trans-11-eicosenoic acid, ethyl linoleate, 7-O-methylisomucronulatol, myricanone, bisdemethoxycurcumin, wallichilide, astrapterocarpan, and kumatakenin. The active ingredients target a series of genes, including *MAPK3*, *MAPK1*, *HSP90AA*1, *STAT3*, *PIK3R1*, *PIK3CA* and *AKT1*, and can potentially regulate the PI3K/AKT, MAPK/ERK, Ras, cAMP and Rap1 signaling pathways to exert a neuroprotective role in patients with ischemic stroke, Qi deficiency and blood stasis syndrome. The cerebral protective effects of each of the drugs that constitutes HCSE have been extensively reported in previous studies; Huangqi promotes the proliferation of neural stem cells, Chuanxiong inhibits inflammation, Sanleng prevents the aggregation of platelets, and Ezhu inhibits autophagy (Huang et al., 2018; Fei Yin et al., 2020; Min Wang et al., 2020; Jia et al., 2021; Wang et al., 2021). In the present study, we found that HCSE effectively reduced cerebral infarcts and the neuronal dysfunction caused by ischemic stroke with Qi deficiency and blood stasis syndrome. KEGG pathway annotation suggested that the active ingredients of the new formulation exert beneficial effects by regulating the PI3K/AKT and MAPK signaling pathways. By analyzing the KEGG enrichment of these targets in ischemic stroke with Qi deficiency and blood stasis syndrome, we found that the PI3K/AKT and MAPK signaling pathway were the main pathways involved. Thus, by regulating the PI3K/AKT and MAPK signaling pathways, it may be possible to improve Qi and blood in patients with ischemic stroke.

The PI3K and MAPK signaling pathways are abnormally regulated in cerebral ischemia; by modulating these pathways, it is possible to alleviate the neuronal injury caused by ischemia (Tu et al., 2015). Research has also shown that HSP90 inhibitors protect against ischemia-induced neural progenitor cell death via the PI3K/AKT and MAPK/ERK pathways (Kwon et al., 2008; Wang et al., 2011; Bradley et al., 2014). On the one hand, the PI3K/AKT signaling pathway is known to play key roles in regulating cell proliferation, differentiation, apoptosis, and migration (Samakova et al., 2019) and is critical for neuronal growth and survival following cerebral ischemia (Zhao et al., 2016). Previous studies have reported that modulation of the PI3K/AKT/mTOR pathway upregulates the bcl-2 protein and increases ischemic tolerance in the semi-dark zone, thereby reducing apoptosis, increasing VEGF expression, and promoting cerebral angiogenesis (Hou et al., 2018; Liang et al., 2018). On the other hand, the extracellular signal-regulated kinases MAPK1/ERK2 and MAPK3/ERK1 are members of the MAP kinase family and are involved in cell proliferation, differentiation, transcriptional regulation, and apoptosis. A series of cascade reactions are known to be involved in ischemic stroke (Sun and Nan, 2016). Numerous previous studies have shown that the MAPK/ERK signaling pathway disrupts the blood-brain barrier, affects neurocyte apoptosis, and enhances the expression of neuronal inflammatory factors after ischemic stroke (Irving et al., 2000; Cao et al., 2016; Wang et al., 2019). The downregulation of MAPK1 has been shown to reduce the levels of TNF-α, IL-6, and reactive oxygen species, thereby reducing neuroinflammation, oxidative stress, and neuronal damage (Zhang et al., 2020). In a previous study, Mostajeran found that inhibition of the ERK signaling pathway by U0126 reduced neuronal death and significantly upregulated the expression of Tie-2, thereby promoting post-stroke vascular stabilization and angiogenesis (Mostajeran et al., 2017).

The cascade response of the PI3K and MAPK signaling pathways is regulated by complex feedback and crosstalk mechanisms. Zhou et al. reported negative crosstalk between the MAPK and PI3K/AKT signaling pathways, with AKT inhibiting the MAPK signaling pathway by phosphorylating and inhibiting the Raf1 node during cerebral ischemia (Zhou et al., 2015). This is consistent with the results of the present study, in which phosphorylated AKT levels were significantly lower, and phosphorylated ERK 1/2 levels were significantly higher, in mice suffering from ischemic stroke. Levels of phosphorylated AKT were increased while levels of phosphorylated ERK 1/2 were decreased in mice with ischemic stroke when treated with HCSE. We suggest that HCSE protects against post-ischemic injury not only by reducing ERK activity, but also by increasing crosstalk between AKT and ERK. The reduction in ERK activity exerts cerebral protective effects.

In summary, we used data mining and network pharmacological target prediction to identify a new treatment formulation (HCSE) for patients suffering from ischemic stroke with Qi deficiency and blood stasis syndrome. HCSE significantly reduced infarct volume and improved neurological function. We found that multiple target genes and pathways participated in the action of HCSE against cerebral ischemia stroke. The mechanisms underlying the neuroprotective effects of HCSE were closely related to activation of the PI3K/AKT and MAPK/ERK signaling pathways. Our results provided evidence for the positive effects of HCSE on ischemic stroke with Qi deficiency and blood stasis syndrome. However, further research now needs to verify the mechanisms underlying the action of HSCE on the improvement of cerebral ischemic injury *via* the regulation of the PI3K/AKT and MAPK/ERK pathways; it is possible that other neuroprotective mechanisms are involved. In addition, it is important to investigate the internal mechanisms that link Qi deficiency, blood stasis syndrome and cerebral ischemic injury, as this could provide a more enhanced theoretical basis for the prevention and treatment of stroke with TCM.

## Data Availability

The raw data supporting the conclusion of this article will be made available by the authors, without undue reservation.

## Glossary

AKT: Protein Kinase B
BBB: Blood-Brain Barrier
BC: Betweenness Centrality
BCA: Bicinchonininc Acid
bcl-2: B-Cell Lymphoma-2
BP: Biological Process
BSA: Bovine Serum Albumin
cAMP: Cyclic Adenosine Monophosphate
CC: Cellular Component: Closeness Centrality
CC: Cellular Component: Closeness Centrality
CCA: Common Carotid artery
DC: Degree Centrality
DL: Drug-Likeness
EC: Eigenvector Centrality
ECA: External Carotid artery
ERK: Extracellular Regulated Protein Kinases 1 and 2
FDR: False Discovery Rate
GO: Gene Ontology
HCSE: Huangqi-Chuanxiong and Sanleng-Ezhu
HSP90: Heat Shock Protein 90
HSP90AA1: Heat Shock Protein 90 Alpha Family Class A Member 1
ICA: Internal Carotid artery
ICR: Institute of Cancer Research
IL6: Interleukin 6
KEGG: Kyoto Encyclopedia of Genes and Genomes
MAPK: Mitogen-Activated Protein Kinases
MAPK1: Mitogen-Activated Protein Kinase 1
MAPK3: Mitogen-Activated Protein Kinase 3
MCAO: Middle Cerebral artery Occlusion
MF: Molecular Functions
mTOR: Mechanistic Target of Rapamycin Kinase
OB: Oral Bioavailability
p-AKT: Phospho-Protein kinase B
p-ERK1/2: Phospho-Extracellular Regulated Protein Kinases 1 and 2
PI3K: Phosphoinositide 3-kinase
PIK3CA: Phosphatidylinositol-4,5-Bisphosphate 3-Kinase Catalytic Subunit Alpha
PIK3R1: Phosphoinositide-3-Kinase Regulatory Subunit 1
PPI: Protein-Protein Interaction
Raf1: Raf-1 Proto-Oncogene, Serine/Threonine Kinase
Ras: Rat Sarcoma
SDS-PAGE: Sodium Dodecyl Sulfate Polyacrylamide Gel Electrophoresis
STAT3: Signal Transducer and Activator of Transcription 3
TBS: Tris Buffered Saline
TCM: Traditional Chinese Medicine
Tie-2: Tyrosine-Protein Kinase
TNF-α: Tumor Necrosis Factor-α
TTC: 2, 3, 5-Triphenyl Tetrazolium Chloride
VEGF: Vascular Endothelial Growth Factor
